# The effect of a web-based lifestyle intervention on nutritional status and physical activity on prevention of COVID-19: a randomized controlled trial in women's empowerment

**DOI:** 10.3389/fnut.2023.1172014

**Published:** 2024-01-19

**Authors:** Farhad Pourfarzi, Aziz Kamran, Maryam Zare, Jafar Mohammadshahi

**Affiliations:** ^1^Digestive Disease Research Center, Ardabil University of Medical Sciences, Ardabil, Iran; ^2^Health Education and Promotion, School of Medicine and Allied Medical Sciences, Ardabil University of Medical Sciences, Ardabil, Iran; ^3^Department of Nutrition, Khalkhal University of Medical Sciences, Khalkhal, Iran; ^4^Infectious Disease, School of Medicine, Ardabil University of Medical Sciences, Ardabil, Iran

**Keywords:** lifestyle, healthy diet, physical activity, COVID-19, web-based

## Abstract

**Background:**

Healthy dietary intake and physical activity affect the immune systems. The present study aimed to investigate the effects of a web-based lifestyle intervention on nutritional status, physical activity, and prevention of COVID-19.

**Methods:**

Three hundred-three women (30–60 years old), who did not have COVID-19 in the City of Ardabil, participated in this study. Participants were randomized into an intervention (*n* = 152) or control group (*n* = 151). The intervention group received eight online educational sessions focusing on a healthy diet and physical activity via the website. There was no educational session for the control group during the intervention, but they were placed on the waiting list to receive the intervention and given access to the website and educational content after the follow-up. Outcomes were nutritional status, physical activity, immunoglobulin G (IgG), and immunoglobulin M (Ig M) antibody titers against the virus. They were evaluated at the baseline, after 4 and 12 weeks.

**Results:**

Significant improvements in weight (*P* < 0.001), BMI (*P* < 0.001), total energy (*P* = 0.006), carbohydrate (*P* = 0.001), protein (*P* = 0.001), and fat (*P* < 0.001) were found for the intervention group compared to the control group during the study. MET-min/week for moderate physical activity increased during the time for the intervention and control groups (*P* < 0.001 and *P* = 0.007, respectively). MET-min/week for walking activity rose in the post-intervention and follow-up compared to that in the baseline in the groups (*P* < 0.001 for both groups). Total physical activity was increased during the study (*P* < 0.001) for both groups. The mean of serum IgG and IgM titers against the virus were increased during the study in both groups in time effect (*P* < 0.001). There was a significant time x group interaction for carbohydrate and fat intakes (*P* = 0.005 and *P* = 0.004, respectively).

**Conclusion:**

The web-based lifestyle intervention may improve nutritional status and physical activity, and have the potential to reduce the risk of contracting a COVID-19 infection.

## Introduction

Coronavirus 2019 disease (COVID-19) was diagnosed as a pandemic by the World Health Organization (WHO) in March 2020, which has led to economic, public health, and social crisis (1). At the time of writing this article, there are more than 676 million coronavirus cases and the total deaths are more than six million worldwide. There have been more than 7 million COVID-19 cases and more than 144,000 deaths in Iran.^1^ The clinical symptoms vary from fever, headache, sore throat, dry cough, and fatigue to progressed symptoms, including pneumonia and death (2). The progress of COVID-19 disease is associated with a rise in inflammatory cytokines and IgG and IgM. Therefore, detecting IgG and IgM antibodies has been more consistent than the nucleic acid detection assay. This method is cheap and simple. This method may play an important role in the diagnosis and epidemic control (3, 4). COVID-19 can be transmitted from person to person. It threatens human health, especially in vulnerable populations, such as women, children, and the elderly; moreover, vulnerable people are at a higher risk of infection (5, 6). Women include the majority of health-related roles. The development of gender-equitable disaster response and reconstruction results from the empowerment of women. Thus, gender remains a basic consideration in infectious disease and during pandemic planning and response. A group at risk during natural disasters and social crises are women. In the majority of societies, women play an important role as health liaisons in changing behaviors and controlling pandemics (7). Governments have put into practice certain strategies to prevent the spread of COVID-19 (8). Unfortunately, the COVID-19 lockdown and social distancing have affected people's lifestyles, especially nutrition patterns, and physical activity in the whole world (9). A sedentary lifestyle and poor eating behavior have increased, which is associated with numerous disorders (10). In accordance with individuals' lifestyles, women are the least active and spend more time watching TV than men (6, 7).

Physical activity training was proven to be one of the most effective lifestyle interventions capable of preventing metabolic disturbances and improving the inflammatory state (11). Physical activity affects the immune system, so its moderate practice boosts the body's immune response, reducing the incidence and severity of infectious processes, especially respiratory diseases (12). COVID-19 lockdown has caused a reduction in physical activity by 36.4% in adults. House confinement increases the consumption of unhealthy food (13). Studies have indicated that the diet during the lockdown includes further energy intake compared to that before COVID-19 (9, 14, 15). Healthy dietary intake affects the immune system and health outcomes during the COVID-19 pandemic (16). A balanced diet strengthens the immune system in response to infection and reduces the severity and complication of COVID-19 disease (17). Nowadays, unhealthy dietary habits have increased in most countries, and poor eating behavior is associated with a higher risk of diseases. There is an urgent need to improve the quality and eating behavior of humans (18). An active lifestyle with an increased level of physical activity affects the immune system (19). Meanwhile, physical activity enhances immune surveillance (20). Based on guidelines, adults are recommended to do 150 min/week of moderate to vigorous physical activity to prevent diseases (21). Therefore, lifestyle interventions increasing physical activity and improving nutritional status are of great necessity during the period of social distancing caused by the disease's pandemic; these strategies are effective in the management of chronic diseases (22). In addition, it seems that due to the widespread complications and high mortality rate of COVID-19 disease, nutrition and physical activity education is essential in strengthening immunity. Nowadays, the number of internet and smartphone users has increased, as a result of which electronic, virtual, and mobile health intervention programs are growing worldwide (23). Under this circumstance, for the prevention of COVID-19 spread, web-based lifestyle intervention would be beneficial and cost-effective. There is a lack of evidence of the effects of a web-based lifestyle intervention to prevent COVID-19.

Thus, we hypothesized that the women who receive lifestyle intervention strategy web training strategies will be more likely to develop a healthy diet and physical activity, and will be less increasing IgM or IgG more than 1.1, will be less likely to develop COVID-19 than the control group. Herein, we conducted a web-based lifestyle intervention strategy in order to evaluate the effectiveness of women's empowerment in terms of a healthy diet and physical activity to prevent COVID-19.

## Methods

### Study protocol

This study was designed as a parallel randomized controlled trial and single-blind and was conducted over 6 months. The last participants were recruited on 20 January 2021. This study had a 3-month follow-up until 21 May 2021. This research was conducted according to the guidelines laid down in the Declaration of Helsinki, and received the approval of the research ethics committee of Ardabil University of Medical Sciences and the clinical trial, IR.ARUMS.REC.1399.284, Approval code Irct.ir: IRCT20221228056969N.

Participants who completed and signed a written informed consent form participated in this study.

### Study population

**Figure 1:**
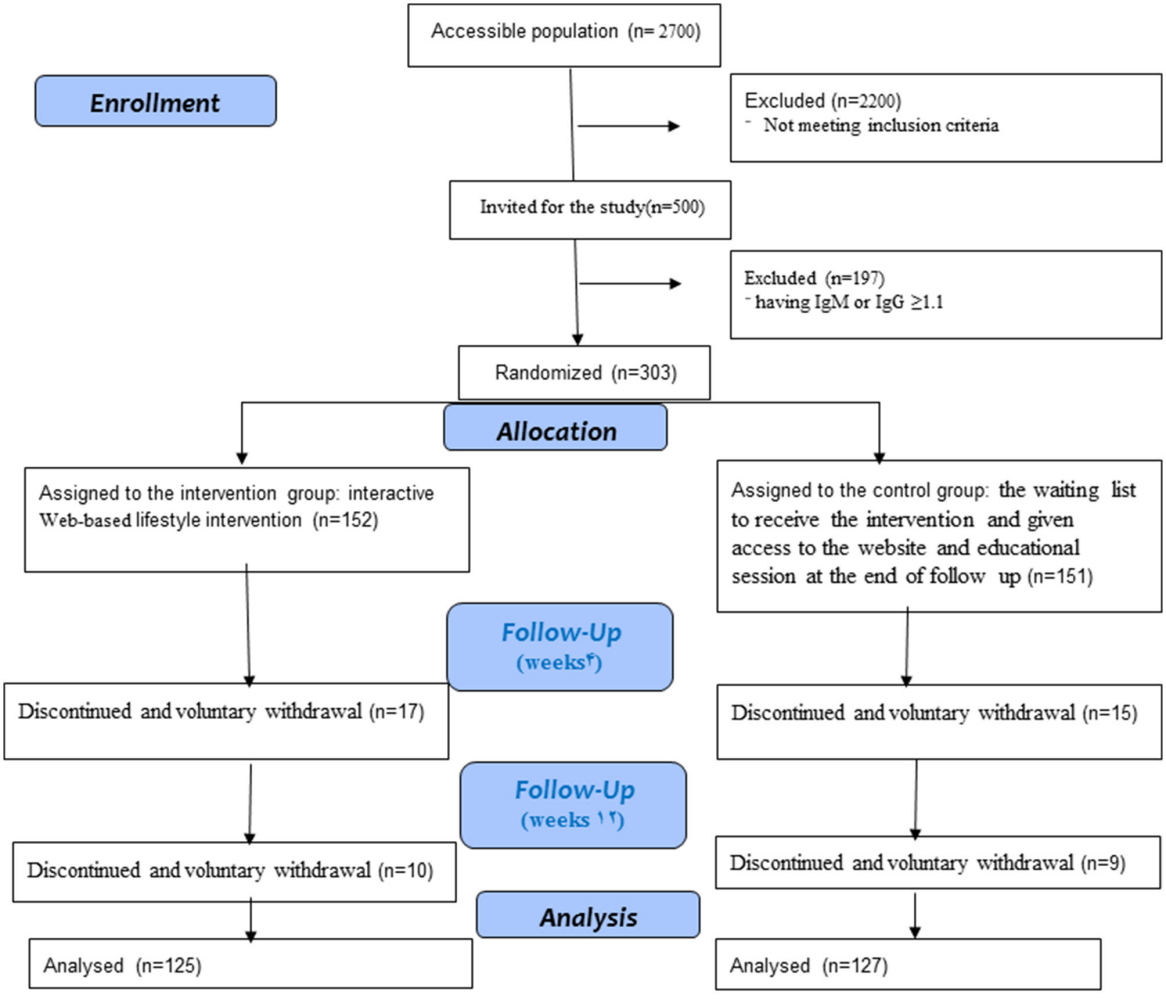
CONSORT flow diagram of lifestyle intervention during COVID-19 pandemic.

Admission began among the healthcare centers in Ardabil, Iran. Ardabil province is located in the northwest of Iran, with a population of over 1 million and 300 thousand people who speak Azeri, and it is divided into five regions. Ardabil, the capital of Ardabil province, was chosen for this study (24). Participants were contacted via a telephone call and screened for the inclusion criteria. Finally, the eligible interested individuals were invited to a free assessment performed by the staff of the Digestive Disease Research Center (DDRC). The women were screened in the baseline by an examination test. Randomization was performed after the baseline with Random Allocation Software (RAS). The population that had inclusion criteria was 500 participants, 197 of whom had IgM or IgG ≥1.1. According to the kit manufacturer guidelines, the cut-off index was calculated, where IgG titers and Ig M titers ≤1.1 were negative and IgG titers and Ig M titers ≥1.1 were positive. Therefore, 303 participants were randomized into two groups, the intervention group (n = 152) and the control group (*n* = 151), by a researcher. Figure 1 shows the study process. Because of the nature of this study, only the analysts were masked in group allocation.

The eligible participants were women (30–60 years of age); literate; having IgM or IgG ≤1.1 at baseline of the study; having access to the Internet, a computer, or smartphone; having the necessary skills to work with the Internet; and having consented to participate in this investigation. The exclusion criteria were having a history of chronic disease, being pregnant or breastfeeding, having IgM or IgG ≥1.1 (which indicates that the person had COVID-19) prior to the intervention, and being vaccinated individuals.

### Interventions

All participants, the intervention and control groups, received information about how to work Big Blue Button and a website designed by a researcher.

The intervention group was given access to a WhatsApp mobile group and website by code access. The mobile app was created to coordinate virtual classes. It allows the users to review their weekly class plans. Web developers and graphic designers created a professional, attractive, and user-friendly website with the address https://edusarscov.com. The website includes a home page, instructions, online and offline classes, and contacts us. Before the beginning of the intervention, the researcher explained the intervention objectives and sessions in the mobile app group. Four sessions of training courses on healthy diet were held once a week. Physical activity sessions were held for four sessions, once a week. This intervention program was conducted via virtual courses through Big Blue Button in the online class section on the website for the intervention group. The details of the class of the session on a healthy diet and physical activity are provided in Supplementary material (23, 25, 26). A number of researchers specializing in healthy diet and physical activity held online classes. All the educational content of the course was uploaded in the offline class section on the website. At the end of the intervention, an online test was conducted to evaluate the participants.

The participants in the control group had a meeting with DDRC employees in the baseline assessments. After the assessments in the baseline, 4 weeks (post-intervention), and 12 weeks (follow-up), the control subjects were offered the intervention and given access to the website and educational content. Actually, there was no educational session for the control group during the intervention, but they were placed on the waiting list to receive the intervention.

### Measures and outcomes

#### Socio-demographic

The General Information Questionnaire was administered at the baseline to collect the following information: age (year), monthly income (million Iranian Rials, IRR), family history of COVID-19, and having a particular diet.

#### Outcome measures

Blood pressure (BP) was measured with a digital sphygmomanometer after the subjects were seated at rest for 15 min. The height and body weight of the participants were measured without shoes, and they were dressed in light clothing with a stadiometer (RASA, Manufactured in Iran) to the nearest of 0.1. The body mass index (BMI) was calculated using the body weight in kilograms divided by the square of height in meters (BMI, in kg/m^2^). Blood samples were collected via venepuncture (Ayrik, Iran) 10 ml. Immunoglobulin G (IgG) and immunoglobulin M (Ig M) antibody titers for COVID-19 were detected via enzyme-linked immunosorbent assay, using kits (ELISA; Pishtaz, Iran) according to the manufacturer instructions.

### Physical activity

Physical activity was measured with the short form of the International Physical Activity Questionnaire (IPAQ): a seven-item questionnaire validated in Iran. Finally, the Metabolic Equivalent of Task (MET) was calculated for minutes in the week for each physical activity level (PAL). PAL is classified into three categories, namely the low category (the lowest level of physical activity with MET < 600), moderate category (five or more days of moderate-intensity activity and/or walking of at least 30 min per day with 600 < MET < 1,500), and high category (1,500 MET-min per week of vigorous-intensity physical activity spread over at least 3 days per week, or 3,000 MET-min per week of moderate to vigorous-intensity physical activity spread over the seven days of the week) (27).

A physical activity behavior questionnaire was also utilized. This questionnaire consists of five items answered as never, sometimes, usually, and always, where responses range from “1”= never to “4” =always. Based on the Likert scale, the minimum score is 5 and the maximum is 20. A score of 5–10 is considered a low score, 10–15 is moderate, and 15–20 is a good score. This questionnaire reliability was established and the Cronbach's alpha of this instrument was 0.821.

### Nutritional status

To assess nutritional status, a 24-h dietary recall was used (28). The subjects were answered to complete a 24-h dietary recall about the food items that they consumed during the 24 h preceding the interview. Household handy measures were taken to aid the subjects in the estimation of the portion size of their food intake and beverage and the portions were converted into grams. Data on a 24-h dietary recall as grams were entered in Nutrition 4 (N4), a computer program, and the levels of total energy (kcal), carbohydrate, protein, and total fat intake were calculated.

A 14-item healthy eating behavior questionnaire was developed by the authors to assess healthy eating. The Cronbach's alpha of this tool was.617, which shows it is a reliable tool.

The subjects were asked to rate their responses on a four-point scale as never, sometimes, usually, and always, with scores ranging from “1”= never to “4” =always. The scores on Likert scoring ranged from 14 to 56 points. A score of 14–28 was low, 28–42 was moderate, and 42–56 was good eating behavior.

### Statistical analysis

The sample size was set considering a type 1 error of 0.05, type 2 error of 0.20, and success rate of p1 = 0.25 and p2 = 0.40; the minimum required sample size was 101 in each group. Considering the sample loss rate of about 50%, the minimum final sample volume in each group will be about 151 people in each group (29).

All the statistical analyses were performed using SPSS software version 25.0 (IBM, Chicago, Illinois, USA). The obtained data are shown as (mean standard deviation) and frequency (percentage) for quantitative and qualitative variables, respectively. The normality of data distribution was assessed using the Kolmogorov–Smirnov test. The Chi-square test (or Fisher exact test) was employed to compare qualitative factors between the two groups. An independent sample t-test was used to compare quantitative variables among the groups. Cochran's *Q* test was utilized to determine if there are differences concerning the dichotomous dependent variables between the groups across time. Through the use of the repeated measures ANOVA and Friedman test, continuous data in groups were evaluated. Mann-Whitney test compared the mean outcome quantities between the two groups in each time studied. To eliminate the effects of the confounding factors, a general linear model (GLM) with generalized estimating equations (GEE) approach was performed to assess the response variables changes by adjusting the confounding variables including age, salary, and family history. A *P* < 0.05 was considered to be statistically significant.

## Results

### Baseline data

Most women in both groups were between the ages of 40–50 years. In the intervention group, 41.3% of the subjects, and in the control group, 52.3% of them had a monthly income range of 10–20 million Iranian Rials (IRR). COVID-19 infection was observed in the family history of 52% (34.2%) and 35(23.2%) of the participants in the intervention and control groups, respectively. The majority of them in both groups did not follow a special diet. The monthly income and family history of COVID-19 were significantly different between the two groups (*P* = 0.019 and *P* = 0.034, respectively) (Table 1).

**Table 1:** Demographic characteristics of participants' intervention and control groups.

		**Study group**		***P*-value**
		**Intervention**	**Control**	
		**Frequency (%)**	**Frequency (%)**	
Age—y	30 ≤	51 (33.6)	48 (32%)	0.163^a^
	40–50	63 (41.4)	59 (39.3%)	
	≥50	38 (25.0)	44 (29.1%)	
Monthly Income—million Iranian Rials—IRR	10–20	62 (41.3)	78 (52.3)	0.019^a^
	20–30	42 (28.0)	39 (26.2)	
	30–40	10 (6.7)	14 (9.4)	
	40–50	15 (10.0)	9 (6.0)	
	≥50	21 (14.0)	9 (6)	
Family history of COVID-19 infection	Yes	52 (34.2)	35 (23.2)	0.034^b^
	No	100 (65.8)	116 (76.8)	
Having a special diet	Yes	2 (1.3)	1 (0.7)	0.623^b^
	No	151 (99.3)	149 (98.7)	

Data are *n* (%). Analyses are ^a^Mann–Whitney, and ^b^Fisher's exact test. There is no significant difference between the study groups in demographic characteristics, except monthly income and family history of COVID-19 infection (*p* < 0.05).

### Outcomes

**Figure 2:**
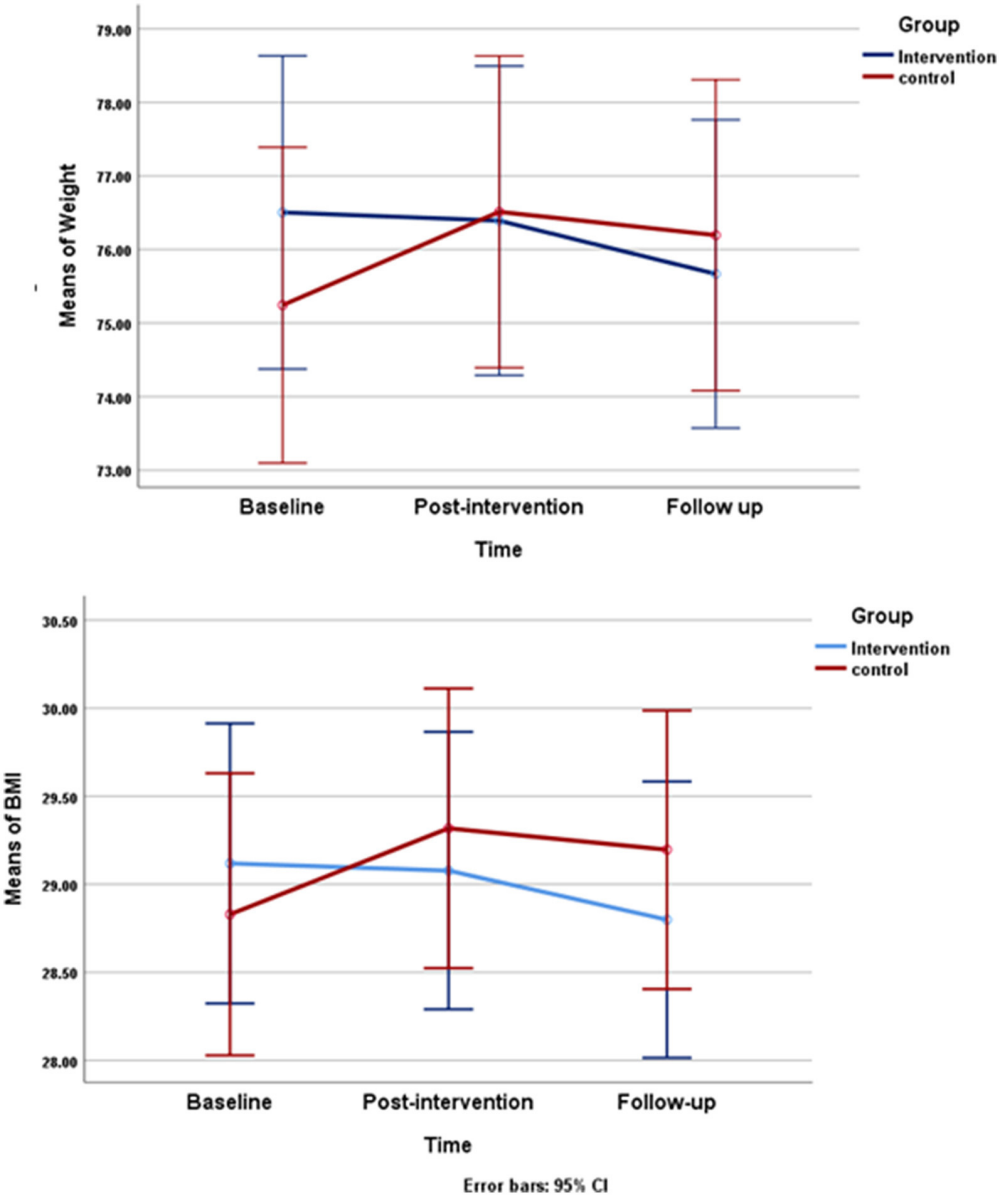
Changes in mean of weight and body mass index (BMI) during study changes in mean of body weight (kg) and changes mean of BMI (kg/m^2^) from baseline through follow up are shown in the control and intervention group. Repeated measures ANOVA indicated that there is a statically significant difference among the three data collection between both of group.

Table 2 depicts the changes in outcome variables from the baseline to follow-up. However, there were no significant differences concerning systolic blood pressure between the two groups. However, the diastolic blood pressure was statistically significant between them (*P* < 0.001). Significant within-group and interaction differences were found regarding weight (*P* < 0.001) and BMI (*P* < 0.001). As shown in the plots, the weight and BMI in the control group were lower at the start of the experiment, but higher in this group in the follow-up compared to those in the intervention (Figure 2). Repeated measure ANOVA showed a significant difference during the time in terms of dietary intake: total energy (*P* = 0.006), carbohydrate (*P* = 0.001), protein (*P* = 0.001), and fat (*P* < 0.001). A significant time x group interaction effect was observed for carbohydrate and fat intakes (*P* = 0.005 and *P* = 0.004, respectively). Therefore, the effect of the treatment on carbohydrates and fat would depend on time (Table 2). We did not find any significant differences in healthy dietary behavior between the intervention and control groups and also during the time.

**Table 2:** Changes in outcome variables from baseline to follow-up.

	**Group**	**Time**			***p*** **-value**		
		**Baseline**	**Post-intervention**	**Follow up**	**Time effect**	**Group effect**	**Interaction effect**
		**Mean (SD)**	**Mean (SD)**	**Mean (SD)**			
**Blood pressure—mm Hg**							
Systolic	Intervention	121 (17)	121 (17)	122 (18)	0.310	0.292	0.310
	Control	118 (13)	119 (13)	119 (13)			
Diastolic	Intervention	85 (21)	85 (20)	85 (20)	0.310	< 0.001	0.310
	Control	76 (11)	76 (12)	77 (11)			
Weight—kg	Intervention	76.10 (12.77)	75.67 (12.81)	75.46 (12.98)	< 0.001	0.893	< 0.001
	Control	74.40 (11.19)	75.93 (11.00)	76.20 (10.89)			
BMI—kg/m^2^	Intervention	28.96 (4.80)	28.84 (4.87)	28.80 (4.80)	< 0.001	0.838	< 0.001
	Control	28.51 (4.12)	29.09 (4.09)	29.20 (4.08)			
**Nutrition intake**							
Total energy-Kcal/day	Intervention	1,995.38 (735.80)	1,779.73 (480.78)	1,938.61 (531.86)	0.006	0.395	0.081
	Control	1,904.54 (727.10)	1,890.38 (623.36)	2,040.40 (716.72)			
Carbohydrate-g/day	Intervention	249.62 (173.01)	188.35 (76.62)	199.77 (80.16)	0.001	0.211	0.005
	Control	233.54 (137.80)	221.81 (107.37)	233.57 (91.80)			
Protein-g/day	Intervention	72.67 (25.63)	71.30 (21.37)	79.76 (23.25)	0.001	0.393	0.190
	Control	74.97 (30.53)	67.85 (19.16)	75.31 (24.59)			
Fat-g/day	Intervention	77.49 (97.86)	48.21 (17.71)	47.20 (23.17)	< 0.001	0.929	0.004
	Control	61.70 (34.99)	55.77 (26.87)	57.82 (29.88)			
Healthy dietary behavior	Intervention	43.8 (5.4)	43.8 (5.3)	44.3 (5.7)	0.316	0.205	0.942
	Control	42.9 (5.2)	43.2 (5.3)	43.8 (5.1)			

Data are means ± SD. Analysis is repeated measure ANOVA. BMI, body mass index is the weight in kilograms divided by the square of the height in meters.

There are no significant differences among the three data collection times relating to vigorous physical activity in both groups. In the intervention group, MET-min/week for moderate physical activity increased during the time (*P* < 0.001). In the control group, MET for moderate activity rose among the three data collection times (*P* = 0.007). In MET-min/week for moderate physical activity, there were significant differences between the intervention and control groups in the post-intervention (*P* = 0.024) and follow-up (*P* = 0.019). The level of walking activity rose in the post-intervention and follow-up compared to that in the baseline in the groups (*P* < 0.001 for both groups). There were no significant differences between the intervention and control groups in terms of walking activity at each time. Total PAL in minutes per week indicated an increasingly significant difference between the three data collections during the study in both groups (*P* < 0.001 for both groups). In total PAL, we detected no significant differences between the intervention and control groups at each of the three data collection times. Finally, the result of the Friedman test and Mann–Whitney test in the intervention and control groups did not indicate any significant differences during the study, neither in the groups nor between them, in terms of physical activity behavior (Table 3).

**Table 3:** Physical activity level and physical activity behavior among participants within three data collection times.

	**Group**	**Time**						***p*-value^†^**
		**Baseline**		**Post-intervention**		**Follow up**		
		**Mean (SD)**	**Median (IQR)**	**Mean (SD)**	**Median (IQR)**	**Mean (SD)**	**Median (IQR)**	
**Physical activity level MET-min/week**								
Vigorous	Intervention	23.7 (209.9)	0 (0, 0)	26.7 (22.7)	0 (0, 0)	13.4 (97.8)	0 (0, 0)	0.368
	Control	22.5 (145.9)	0 (0, 0)	23.2 (152.5)	0 (0, 0)	46.9 (219.4)	0 (0, 0)	0.819
*p*-value^‡^		0.312		0.487		0.082		
Moderate	Intervention	644.3 (933.2)^ab^	240 (120, 720)	989.5 (1,179.9)^b^	480 (240, 1,440)	972.9 (1,114.8)^a^	480 (240, 1,440)	< 0.001
	Control	574.9 (873.4)	240 (120, 720)	681.7 (922.8)	390 (160, 960)	661.8 (930.1)	240 (160,720)	0.007
*p*-value^‡^		0.427		0.024		0.019		
Walking	Intervention	149.1 (246.9)^ab^	33 (0, 198)	215.38 (365.3)^a^	99 (0, 297)	310.2 (493.8)^b^	132 (33, 396)	< 0.001
	Control	159.6 (295.4)^a^	33 (0, 198)	238.40 (534.4)	82.5 (0, 198)	299.8 (555.5)^a^	132 (16.5, 396)	< 0.001
*p*-value^‡^		0.785		0.635		0.642		
Total physical activity level	Intervention	817.1 (1,020.5)^ab^	537 (201.2, 939)	1231.5 (1,296.1)^a^	753 (278, 1,836)	1,296.6 (1,282.5)^b^	876 (339, 1,986)	< 0.001
	Control	757.1 (949.0)^a^	480 (198, 852)	943.3 (1,099.7)	568.5 (249.5, 1,212)	1,008.6 (1,086.9)^a^	678 (273, 1,356)	< 0.001
*p-*value^‡^		0.614		0.058		0.105		
Physical activity behavior	Intervention	8.8 (3.2)	10.0 (6.0, 10.0)	8.8 (3.2)	10.0 (6.0, 10.0)	8.8 (3.1)	10.0 (6.0, 10.0)	0.834
	Control	8.4 (2.5)	8.0 (6.0, 10.0)	8.4 (2.5)	8.0 (6.0, 10.0)	8.5 (2.6)	8.0 (6.0, 10.0)	0.459
*p*-value^‡^		0.568		0.429		0.746		

Data are means ± SD and median ± IQR.

^†^Analyses are Freidman test.

^‡^Mann–Whitney test.

^a, b^Similar letters show statistically significant *P* < 0.05.

MET, denotes metabolic equivalents task; IQR, interquartile range.

There was no significant difference between the intervention and control groups in terms of characteristics of plasma antibodies and the means of plasma antibody titers against COVID-19. The result of the Cochran's Q test revealed a statistically significant difference in the intervention group concerning IgG against the virus (*P* = 0.002). Moreover, it indicated a statistically significant difference in the control group in terms of Ig G and Ig M against the virus (*P* < 0.001 and *P* = 0.023, respectively) (Table 4).

**Table 4:** Characteristics of plasma antibodies in participants within three data collection times.

**Group**			**Time**			***p*-value^†^**
			**Baseline**	**Post-intervention**	**Follow up**	
			**Frequency (%)**	**Frequency (%)**	**Frequency (%)**	
Intervention	IgG (Binned)	< 1.1	152 (100%)^a^	135 (100%)^b^	119 (95.2%)^ab^	0.002
		≥1.1	0 (0%)	0 (0%)	6 (4.8%)	
Control	IgG (Binned)	< 1.1	151 (100%)^ab^	136 (98.6%)^b^	116 (91.3%)^a^	< 0.001
		≥1.1	0 (0%)	2 (1.4%)	11 (8.7%)	
*p*-value^‡^			–	0.498	0.222	
Intervention	IgM (Binned)	< 1.1	148 (97.4%)	134 (99.3%)	118 (94.4%)	0.078
		≥1.1	4 (2.6%)	1 (0.7%)	7 (5.6%)	
Control	IgM (Binned)	< 1.1	149 (98.7%)^a^	136 (98.6%)^b^	118 (92.9%)^ab^	0.023
		≥1.1	2 (1.3%)	2 (1.4%)	9 (7.1%)	
*p*-value^‡^			0.684	1.00	0.797	

Data are *n* (%). Analyses are^†^Cochran's *Q-*test and^‡^chi-square (or Fisher's exact test).

^a, b^Similar letters show statistically significant *P* < 0.05.

IgG, immunoglobulin G; IgM, immunoglobulin M, ELISA, enzyme-linked immune sorbent assay antibodies to SARS-CoV-2, determined by ELISA of plasma samples obtained from subjects.

**Figure 3:**
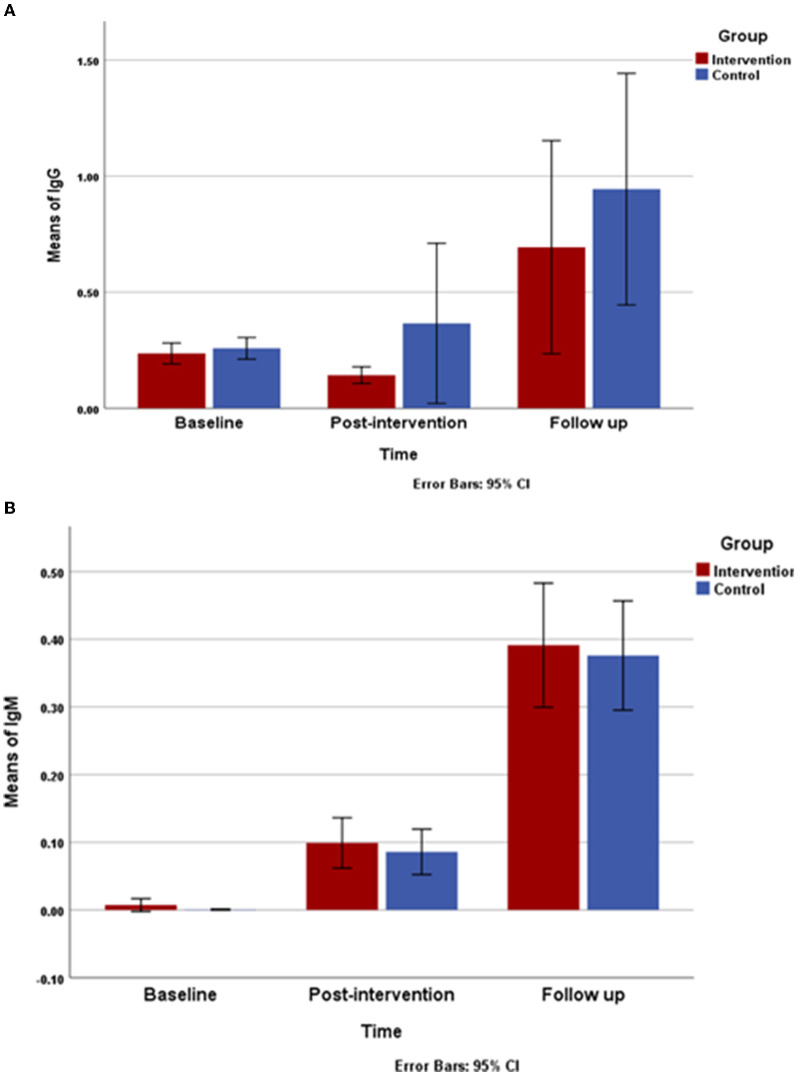
Changes in mean of immunoglobulin G (IgG) and immunoglobulin M (IgM) titers during study **(A)**, changes mean of IgG titers and **(B)**, changes mean of IgM titer from baseline through follow-up are shown in the intervention and control group. Repeated measures ANOVA indicated that there is a statically significant difference among the three data collection between both of group.

The result of repeated measures ANOVA demonstrated an increased mean of serum Ig G and Ig M titers against the virus among the three data collection times in both groups in time effect (*P* < 0.001). However, according to the group and interaction effect, no significant trend was observed in either of the groups in terms of Ig G and Ig M titers against the virus (Figure 3).

Table 5 shows weight, BMI, physical activity behavior, total energy, carbohydrate, protein, fat, and healthy dietary behavior, along with Ig G and Ig M titers changes in the groups by adjusting the effect of confounding variables. After adjusting the confounding variables, age, salary, and family history, there were no significant group differences in terms of weight, BMI, physical activity behavior, total energy, carbohydrates, protein, fat, healthy dietary behavior, and IgG and Ig M titers.

**Table 5:** Evaluation of main outcome changes using a general linear model (GLM) with generalized estimating equations (GEE) approach.

	**Group effect** ^**a**^		**Time effect**				**Interaction (group)** ^*^ **(Time)**			
	**B (CI)**	***p*-value**	**Post-intervention** ^**b**^		**Follow up** ^**b**^		**(Group** = **intervention)** ^*^(time = **post-intervention)**		**(Group** = **intervention)** ^*^(time = **follow up)**	
			**B (CI)**	***p*-value**	**B (CI)**	***p*-value**	**B (CI)**	***p*-value**	**B (CI)**	***p*-value**
Weight—kg	1.1 (−1.5 to 3.7)	0.392	1.2 (1.02 to 1.4)	< 0.001	0.9 (0.6 to 1.3)	< 0.001	−1.4 (−1.7 to 1.2)	< 0.001	−1.8 (−2.3 to −1.2)	< 0.001
BMI—kg/m^2^	0.42 (−0.56 to 1.4)	0.402	0.45 (0.40 to 0.57)	< 0.001	0.36 (0.22 to 0.50)	< 0.001	−0.54 (−0.67 to −0.41)	< 0.001	−0.69 (−0.90 to −0.48)	< 0.001
Physical activity behavior	0.24 (−0.38 to 0.85)	0.442	0.01 (−0.008 to 0.02)	0.286	0.04 (−0.10 to 0.2)	0.615	–	–	–	–
Total energy-Kcal/day	91.8 (−68.9 to 252.6)	0.263	−11.3 (−130.1 to 107.4)	0.852	127.7 (−33.9 to 289.4)	0.122	−206.5 (−377 to −35.9)	0.018	−183.6 (−395.7 to 28.5)	0.090
Carbohydrate-g/day	12 (−23.2 to 47.3)	0.50	−10.8 (−38.9 to 17.3)	0.452	1.1 (−24.3 to 26.6)	0.930	−50.7 (−91.9 to −9.4)	0.016	−51.3 (−90.2 to −12.4)	0.010
Protein-g/day	1.6 (−2.2 to 5.4)	0.409	−4.3 (−8.1 to −0.49)	0.027	3.60 (−0.40 to 7.6)	0.078	–	–	–	–
Fat-g/day	16.1 (−1.4 to 33.6)	0.071	−6.1 (−12.7 to 0.44)	0.068	−4 (−11.2 to 3)	0.260	−23.2 (−40.1 to −6.3)	0.007	−26.2 (−43.1 to −9.3)	0.002
Healthy dietary behavior	0.58 (−0.32 to 1.5)	0.205	0.13 (−0.75 to 1)	0.771	0.66 (−0.23 to 1.5)	0.147	–	–	–	–
IgG Titers	−0.14 (−0.39 to 0.10)	0.248	0.01 (−0.16 to 0.18)	0.913	0.57 (0.24 to 0.90)	0.001	–	–	–	–
IgM Titers	0.01 (−0.03 to 0.06)	0.483	0.09 (0.06 to 0.11)	< 0.001	0.38 (0.32 to 0.44)	< 0.001	–	–	–	–

OR, odds ratio; CI, confidence interval.

^a^Intervention group is compared with the control group (reference).

^b^Post-intervention and follow-up time are compared with baseline.

Analyses are based on general linear model (GLM) with generalized estimating equation (GEE).

## Discussion

The study results revealed a significant difference in terms of diastolic blood pressure between the two groups during the study. The result indicated that the intervention group had a decreasing body weight in the post-intervention (0.271 kg) and the follow-up (0.661 kg) compared with the control group. This study indicated that a lifestyle intervention program could lead to decreased total energy in the post-intervention and the follow-up in the intervention group in comparison with the control group. In the former, carbohydrates decreased in the post-intervention and the follow-up compared to the baseline. Total fat consumption decreased in the post-intervention and the follow-up in the intervention group. Daily protein intake rose during the follow-up in the intervention group. In both groups from baseline through follow-up, the healthy dietary behavior score increased. However, healthy dietary behavior did not indicate significant differences during the study. From the beginning study, in the intervention group, a healthy dietary behavior score was a good score; however, in the control group, the healthy dietary behavior score changed from medium to good scores. The healthy dietary behavior score did not indicate significant differences. Physical activity education did not vary according to the baseline concerning vigorous level. Although the analyses indicated a significant improvement in the post-intervention and follow-up in the majority of physical activity levels in both groups, web-based intervention showed greater improvements (moderate, walking, and total physical activity) in the intervention group than in the control group. The obtained findings did not show a significant modification in the mean score of physical activity behavior. In both groups, the mean score for physical activity behavior was low. In the present study, we found that lifestyle intervention programs in women without COVID-19 infection resulted in a lower risk of getting infected in the intervention group, and the mean of Ig M and IgG against the coronavirus titers increased in the follow-up in both groups.

A previous paper indicated that a web-based intervention on nutritional status, physical activity, and health-related quality significantly decreased systolic and diastolic blood pressure within groups in a patient with metabolic syndrome (22). Our results in terms of blood pressure were contrary to these findings. In 2021, a systematic literature review and meta-analysis yielded a significant decrease in body weight and BMI (30). Our findings are consistent with a recent systematic literature review and meta-analysis. The meta-analysis showed that web-based digital intervention led to greater weight loss in the short term (31). A lifestyle intervention showed that the mean bodyweight of the subjects in the intervention group decreased compared with that in the control group (32). Sevilla et al. conducted lifestyle exercise and nutrition intervention, and body weight did not change after the intervention. Our results in terms of body weight were different from the findings reported by Sevilla et al. (33). We observed a reduction in body weight and BMI. Our findings are consistent with a recent systematic literature review and meta-analysis, showing that multi-component worksite wellness programs improve diet and body weight (30). The present analysis supported previous research implying that web-based intervention, decreased total calorie, fat, and carbohydrate could be of benefit (22). Nevertheless, the dietary behavior included in this study indicated an increased mean score that was not statistically significant. In line with the current study, Sevilla et al. reported that nutrition and adherence to the Mediterranean diet were effective (33). In a cross-sectional study conducted on undergraduate nursing and medical students, a 10-index scale increment of digital healthy diet literacy was associated with increased healthy eating behavior in students (34). However, social distancing during the COVID-19 pandemic has had an impact on individuals' behaviors, reducing the level of physical activity and worsening dietary habits (35); therefore, lifestyle intervention is necessary during the pandemic. A systematic review and meta-analysis concluded that the intervention increased the physical activity of the participants in vigorous and moderate physical activities (36). According to a systematic review, when moderate- to vigorous-intensity physical activity is >150 min/week, it could prevent weight gain (37). Poppe's study reported an increase in moderate and moderate to vigorous intensity levels of physical activity (38). A randomized controlled trial indicated that an educational intervention increased physical activity during the COVID-19 pandemic (33). The results herein are in accordance with those of previous investigations.

Several possible explanations could be considered for the decreased weight and BMI in the intervention group. The main reason is the decrease in carbohydrate and total fat consumption in this group in comparison with the control group. Further increase in physical activity level in the intervention group compared to that in the control group is another reason behind weight loss and BMI. The growth in the level of physical activity and reduction in certain macronutrients in the intervention group compared to those in the control group was using proper educational curriculums in the intervention group. Despite social distancing and mobility restrictions, intensive physical activity had an increasingly significant effect on both groups and the intervention group had a MET-min/week closer to 1,500 MET-min; this score is categorized as a moderate level of physical activity (27). Greater improvement in the total physical activity in the intervention group may be owing to the effectiveness of the web-based intervention. Of course, vaccination started worldwide at the time of the follow-up of this study, and the decrease in home quarantine caused all levels of physical activity to increase in the control group in the follow-up compared to the baseline.

The total energy intake restriction, increased protein intake, and physical activity might play a role in the changes in adipose cells' size and affect anti-inflammatory (11). Obesity affects immunity and there is a relationship between obesity and various infectious diseases. Obesity causes mild chronic inflammation violating innate immunity and adaptation (39). Physical activity affects the immune system and its anti-viral defenses. Several mechanisms interfere with the effect of exercise on cytokines, the increase in physical activity is related to the reduction in fat mass and subsequently a decrease in adipokine secretion and induction of an anti-inflammatory effect through releasing cytokines from contracting skeletal muscle (40). There is the concept of the inverted J theory, where moderate exercise, such as walking, reduces susceptibility to infection, and prolonged, high-intensity exercise increases it (41). In our study, moderate physical activity and walking indicated a further increase in the intervention group than in the control group. In general, the level of physical activity was moderate in the present study, which contributed to boosting the immune response (42). Owing to the importance of the spread of worldwide pandemics, especially COVID-19, lifestyle interventions must be effective and available to vulnerable populations. The Internet, smartphone, and technology programs prepare opportunities for implementing lifestyle interventions (22).

In the current study, we used modern technologies, such as web-based lifestyle intervention strategies for women who are among the high-risk populations. The participants had similar social and cultural (socio-cultural) characteristics because they lived in a province with the same socio-cultural characteristics. Herein, we observed favorable results. These findings indicated that web-based lifestyle intervention could effectively improve body weight, BMI, total energy and carbohydrate intakes, total fat, and protein consumption (total and moderate physical activity, and walking) levels, and healthy dietary behavior scores strengthen the immune response led to a lower prevalence of COVID-19 in the intervention group.

The strengths of the present study include a randomized control trial, large sample size, three months of follow-up, the use of educational approaches, such as PowerPoint, and the use of web-based lifestyle intervention. However, this investigation has certain limitations. Using 24-hour recall dietary in data collection, the error measurement was unavoidable. Accordingly, the intervention programs were relatively short so that they would not overlap with the vaccination process.

## Conclusion

Our results give support to the effectiveness of interactive web-based lifestyle programs in improving weight, BMI, nutritional status, and physical activity which can be effective in boosting immunity and could help prevention of COVID-19. The integration of interactive web-based programs into primary health care practices such as prevention of the pandemics, especially COVID-19, offers possibilities for on-time interaction in a high-risk population with several advantages for administrators of the preventive strategies. Furthermore, the findings of the current study show that web-based lifestyle interventions could be considered beneficial for decreasing the risk of chronic diseases, particularly in vulnerable populations. However, further research is required to corroborate these findings and apply newer technology in the prevention of pandemics.

## Data availability statement

The datasets presented in this article are not readily available because restrictions apply to the availability of some or all data generated or analyzed during this study to preserve patient confidentiality or because they were used under license. The corresponding author will on request detail the restrictions and any conditions under which access to some data may be provided. Requests to access the datasets should be directed to MZ, zaremaryam119@gmail.com.

## Ethics statement

The studies involving humans were approved by AIR.ARUMS.REC.1399.284, Approval code Irct.ir: IRCT20221228056969N. The studies were conducted in accordance with the local legislation and institutional requirements. Written informed consent for participation in this study was provided by the participants' legal guardians/next of kin.

## Author contributions

MZ designed the study, secured the funding, designed the curriculum training strategy of trial and site education coordinated the study, analyzed data, and drafted the manuscript. AK designed the study, designed the training strategy content of the trial and held training for the class, and drafted the manuscript. FP coordinated the study and drafted the manuscript. JM contributed to the design of the trial and drafted the manuscript. All the authors have made an essential contribution to the article.

## Footnotes

^1^ https://www.worldometers.info/coronavirus
