# Supplementary material for: The effect of a web-based lifestyle intervention on nutritional status and physical activity on prevention of COVID-19: a randomized controlled trial in women's empowerment
Source: Front Nutr. 2024 Jan 19;10:1172014. doi: 10.3389/fnut.2023.1172014 (PMC10834691; doi:10.3389/fnut.2023.1172014)
Supplement: Supplementary file 1 [file Data_Sheet_1.doc]

| **Healthy Diet** |
| --- |
| Session 1: The intervention group was educated about a healthy diet in improving the immune system during the COVID-19 pandemic. They were taught components of a healthy diet, overweight, and obesity, and the effect of obesity and weight loss on the immune system. Components diet included carbohydrates, protein, fat, vitamins, and minerals. |
| Session 2: Intervention group was educated about obesity and its effects on inflammation body. The intervention group was encouraged to consume a healthy diet and use of food plan. This session described the benefits of a healthy diet based on balance, moderation, and variety of food. |
| Session 3: Participants of the intervention group were taught My Plate. They were educated food groups include, including bread and cereal, vegetables and legumes/beans, fruit, dairy, and meats. |
| Session 4: Women intervention group was encouraged to plan for food supply, shop for healthy food, and reduce salt, sugar, and fat intake. In this session, women were taught nutritional recommendations for preventing and treatment of COVID-19 disease. |
| **Physical activity** |
| Session 1: Women's intervention group was taught physical activity and immunity. They educated about Participants were taught the importance of regular sports, repetition, and duration of exercise. |
| Session 2. The intervention group was educated about obesity and exercise, the effects of physical activity on immunological, and biochemical parameters, and quality of life. |
| Session 3: Intervention group was taught about the purpose and instructions for the exercise. Women in this group trained easy exercise to an active lifestyle. |
| Session 4: Intervention group was educated on reasons to do exercise. They were educated about the best type of exercise and the proper time for doing exercise. They were asked to be aware of sensations, and thoughts. The intervention group was educated on types of exercise such as aerobic, and resistance training. They were encouraged to exercise during the COVID-19 by following the instruction. |

BOX1. Session Box of a healthy diet and physical activity
